# Assessment of Dietary Mercury Intake and Blood Mercury Levels in the Korean Population: Results from the Korean National Environmental Health Survey 2012–2014

**DOI:** 10.3390/ijerph13090877

**Published:** 2016-09-01

**Authors:** Seong-Ah Kim, YoungMin Kwon, Suejin Kim, Hyojee Joung

**Affiliations:** 1 Department of Public Health, Graduate School of Public Health, Seoul National University, Gwanak-gu, Seoul 08826, Korea; ksacute@snu.ac.kr; 2 Korean National Institute of Environmental Research of the Ministry of Environment, Seo-gu, Incheon 22689, Korea; u11023@korea.kr (Y.M.K.); suenier@korea.kr (S.K.)

**Keywords:** Korean National Environmental Health Survey, mercury, methylmercury, diet, blood

## Abstract

From a public health perspective, there is growing concern about dietary mercury intake as the most important source of mercury exposure. This study was performed to estimate dietary mercury exposure and to analyze the association between mercury intake and blood mercury levels in Koreans. The study subjects were 553 adults, comprising a 10% representative subsample of the Korean National Environmental Health Survey (KoNEHS) 2012–2014, who completed a health examination, a face-to-face interview, and a three-day food record. Dietary mercury and methylmercury intakes were assessed from the three-day food record, and blood mercury concentration was measured using a mercury analyzer. The association between dietary mercury intake and blood mercury levels was analyzed by comparing the odds ratios for the blood mercury levels above the Human BioMonitoring (HBM) I value (5 μg/L) among the three groups with different mercury intakes. The average total mercury intake was 4.74 and 3.07 μg/day in males and females, respectively. The food group that contributed most to mercury intake was fish and shellfish, accounting for 77.8% of total intake. The geometric mean of the blood mercury concentration significantly and linearly increased with the mercury and methylmercury intakes (*p* < 0.001). The odds ratios for blood mercury levels above the HBM I value in the highest mercury and methyl mercury intake group were 3.27 (95% Confidence Interval (CI) 1.79–5.95) and 3.20 (95% CI 1.77–5.79) times higher than that of the lowest intake group, respectively. Our results provide compelling evidence that blood mercury level has a strong positive association with dietary intake, and that fish and shellfish contribute most to the dietary mercury exposure.

## 1. Introduction

There is growing concern about exposure to mercury from a public health perspective [1]. Mercury exposure can have harmful effects on the neurological, cardiovascular, reproductive, and immune systems [2,3]. In particular, because fetuses may be even more sensitive to mercury than adults [4,5], mercury exposure during pregnancy is an important public health concern.

In the general population, potential sources of mercury exposure include the inhalation of mercury vapor in the air, ingestion of foods and drinking water contaminated with mercury, and exposure to dental amalgam through dental care [6]. After occupational exposure, dietary intake is the most important source of mercury exposure, with fish and other seafood as the dominant source of mercury in the diet [7]. Therefore, it is important to estimate dietary mercury intake for an accurate risk assessment of mercury exposure.

Many countries are currently conducting national biomonitoring programs; e.g., the National Biomonitoring Program (NBP) in the United States, the German Environmental Survey (GerES) in Germany, and the biomonitoring of environmental chemicals in the Canadian Health Measures Survey (CHMS) of Canada [8,9,10]. Korea also has a national biomonitoring program, the Korean National Environmental Health Survey (KoNEHS) conducted by the Korean National Institute of Environmental Research (NIER) of the Ministry of Environment (MOE) since 2009. Fish and shellfish are a major part of the Korean diet because Korea is surrounded by ocean on three sides, and thus, fish products account for about 20% of all animal food consumption in terms of energy intake [11]. According to the Korean food balance sheet, fish consumption in Korea is much higher than other countries, including Japan, an island country [11]. Fish and shellfish are the dominant sources of mercury exposure and, therefore, the Korean population faces possible high risk of mercury exposure through food ingestion. In this regard, the Korean population is an appropriate group to assess for mercury exposure via food.

National biomonitoring programs typically include a dietary survey to measure dietary exposures to hazardous materials. However, the dietary assessment methods used in these programs vary depending on the target materials, level of precision and accuracy required for the measurement, time period of interest, and research constraints such as financial resources, time, staff, and respondent characteristics [12]. Generally, a food frequency questionnaire (FFQ) is the most widely used in biomonitoring programs because it is relatively inexpensive, easy to conduct and can reflect long-term dietary intake; however, their quantitative measurements are not highly accurate. On the other hand, open-ended dietary assessment methods, such as food records and 24-h recall, are more accurate for a specific time period, but have rarely been used in large-scale surveys because of the high cost and the respondent burden [12]. Usual intake is not commonly assessed this way due to these challenges, but it can be estimated by open-ended dietary assessment when conducted over three non-consecutive days [13]. Assessing dietary intake of specific materials like mercury by food consumption data can be estimated provided that a mercury database for common foods is available.

A positive relationship between dietary mercury intakes and mercury exposure levels in the blood, urine, and breast milk has been reported elsewhere [14,15,16]. These studies found that the intake frequency of certain food items (mainly fish and shellfish) is positively associated with the mercury levels of a biomarker. Recently, the data form the European project DEMOnstration of a study to COordinate and Perform Human biomonitoring on a European Scale (DEMOCOPHES) showed a clear correlation between the consumption of fish and marine products and mercury levels in the hair [17]. Similarly, several studies conducted in Korea also have shown a relationship between diet and blood mercury concentration; however, these studies were very limited in terms of a small sample size for the population and foods [18,19]. Very few studies have estimated the association between dietary intake of mercury and the exposure levels of biomarkers using three-day food records.

Since the mercury content in food varies according to the region and the natural environment, and the dietary pattern of people is different from country to country, it is required to estimate dietary mercury exposure of Koreans based on the their own distinct dietary characteristics. In this study, we estimated dietary mercury exposure using three-day food records, and analyzed the association between dietary mercury exposure and blood mercury levels in Koreans considering their high consumption of fish and shellfish.

## 2. Materials and Methods

### 2.1. Study Design and Subjects

We used data from the KoNEHS 2012–2014 to estimate the dietary mercury intake of a Korean population. KoNEHS is a cross-sectional, population-based human biomonitoring study of a representative sample of the population (i.e., 6000 adults over 19 years of age) in South Korea that is conducted every three years by the Korean NIER of the MOE. KoNEHS is designed to collect reference data regarding the exposure levels to 21 environmentally hazardous substances for the general population. KoNEHS consists of a health examination survey, a face-to-face interview to determine environmental contamination and exposure factors (such as the residential environment, living conditions, exposure to contaminants at work, lifestyle, and dietary habits), and bio-sample collection. Hazardous materials such as lead, mercury, cadmium, manganese, arsenic, polycyclic aromatic hydrocarbons (PAHs) metabolites, cotinine, phthalates, bisphenol A, and pyrethroid pesticide metabolites are assessed in the blood or urine. The study subjects were 553 adults, representing a 10% of the subjects in the KoNEHS 2012–2014. The subjects were selected as a representative subsamples including sex, age, and residential area. The subjects completed a health examination, a face-to-face interview and a three-day food record. All procedures for the KoNEHS were approved by the Korean NIER Institutional Review Board, and all subjects provided written informed consent.

### 2.2. Dietary Mercury Exposure

Subjects were given a questionnaire to collect a three-day food record through door-to-door visits 3 to 4 weeks before the implementation of the KoNEHS and were instructed how to record their food intakes, and, if necessary, trained staff members additionally helped them to complete the questionnaires by phone. During the seven days prior to the KoNEHS, they were instructed to record consumed foods and beverages with the amounts using food models and pictures from the guideline materials [20] during three non-consecutive days including two days out of weekdays and one of the weekend day. They brought the completed three-day food records to the survey site on the day of KoNEHS, and a trained interviewer clarified the records by adding any omitted items or amounts. Any incomplete questionnaires were finalized by a follow-up with a phone call to the subjects.

To estimate dietary mercury exposure, we connected food consumption data from the three-day food records with a mercury database for commonly consumed food items among the Korean population, which was developed in a previous study [21]. This database is based on the food names and codes of the Korean National Health and Nutrition Examination Survey (KNHANES), and includes a total of 356 food items, which cover 95.1% of all food consumed by the subjects who completed a 24-h dietary recall during the KNHANES 2007–2009. Details of the mercury database are presented in [21]. Each food item consumed by subjects was linked to the mercury content in that food item to calculate a daily total mercury intake per person. The equation used to calculate the daily dietary mercury intake was as follows:

(1) $$\frac{\sum_{i=1}^{n}[{\left\{Food\;intake\;\left(g\right)\right\}}_{i}\;\times \;\{Mercury\;content\;in\;food\;{\left(\mu g/g\right)}_{i}\}]}{3\;\left(days\;the\;subjects\;carried\;out\;the\;dietary\;record\right)}$$

where *i* represents each food item consumed during the days that subjects completed the three-day food record.

### 2.3. Risk Assessment of Dietary Mercury Exposure of Koreans

We compared the estimated daily dietary mercury intake to the Provisional Tolerable Weekly Intake (PTWI) that was established by the Joint Food and Agriculture Organization of the United Nations (FAO) and the World Health Organization (WHO) Expert Committee on Food Additives (JECFA). The PTWIs for inorganic mercury and methylmercury are 4 μg/kg body weight (bw)/week and 1.6 μg/kg bw/week, respectively. According to the 72nd meeting of JECFA, the PTWI for inorganic mercury is considered to be applicable for determining the dietary exposure to total mercury from foods other than fish and shellfish, whereas, for the dietary exposure to mercury from these foods, only the PTWI for methylmercury should be applied [22].

The mercury database used in this study was developed for the total mercury content in food, and, therefore, the intake of total mercury, rather than inorganic or methylmercury, could be directly calculated. In most non-fish food items, mercury concentration is typically near the detection limits and consists mainly of inorganic species [7]. In contrast, most of the mercury in fish is methylated. Therefore, we compared the total mercury intakes from all food items with the PTWI for inorganic mercury, and methylmercury intakes from fish and shellfish with the PTWI for methylmercury. The corresponding methylmercury contents in fish were calculated by taking into consideration that 90% of the total mercury in fish is found in the methylated form, using the following equation [7,23]:

(2) $$\left[\mathrm{Methylmercury}\;\left(\mu g/g\right)\;in\;fish\right]=0.9\;\times \;\left[Total\;mercury\;\left(\mu g/g\right)\;in\;fish\right]\;\times \frac{M_{MeHg\;}\left(215.62\;g/mol\right)}{M_{THg}\;\left(200.59\;g/mol\right)}$$

We compared the estimated daily intake of total mercury and methylmercury with the PTWI for each form of mercury expressed as the Provisional Tolerable Daily Intake (PTDI). The PTDI was 0.57 μg/kg bw/day for total mercury and 0.23 μg/kg bw/day for methylmercury. We determined the dietary exposure to the total mercury and methylmercury levels by sex, age, residential area, obesity, smoking status, and alcohol drinking status. We estimated the proportion of study subjects in different PTWI (converted to PTDI) levels after they were classified into groups according to the classification variables.

### 2.4. Measurement of the Blood Mercury Concentration

In the KoNEHS, blood was collected from all subjects. The medical technologist used a needle to draw blood from a vein in the antecubital fossa of subjects’ arm. The blood was processed and aliquoted into vials for storage. The vials were then refrigerated or frozen before transport to laboratories. Mercury assays were conducted for whole blood at the Seegene Medical Foundation (Seoul, Korea) with a mercury analyzer (DMA-80: Milestone, Bergamo, Italy).

We classified subjects by their blood mercury concentration using the Human BioMonitoring (HBM) value, which is a health-related biological exposure limit value, and determined the proportion of subjects whose blood mercury levels were above the HBM I value. The German HBM Commission defines two different HBM values: HBM I and HBM II. The HBM I value corresponds to the concentration of a substance in a human biological material below which no adverse health effects are expected [24]. The HBM II value represents the concentration above which there is an increased risk of adverse health effects. The HBM I value for blood mercury is 5 μg/L, while the HBM II value is 15 μg/L.

### 2.5. Statistical Analysis

All statistical analyses were performed with SAS version 9.3 (SAS Institute Inc., Cary, NC, USA). The general characteristics and distribution of the percentage PTDI levels and blood mercury levels of the study subjects were described with the chi-square test. Blood mercury levels were presented as the geometric mean (GM) with 95% confidence intervals (CIs). The dietary exposure levels for total mercury and methylmercury by sex, age, and residential area categories were compared by one-way analysis of variance (ANOVA).

To test the linear trends in the blood mercury concentration with dietary mercury intake levels, we used generalized linear model analysis. The subjects were divided into three groups according to their mercury intake. We also calculated the contribution of 18 food groups to the total mercury intake, and analyzed the association between the amounts consumed from specific food groups and the blood mercury concentration.

Finally, we analyzed the association between the dietary mercury intake and blood mercury levels using logistic regression analysis, by comparing the odds ratios and 95% CIs for blood mercury levels above the HBM I among the three groups with different mercury intakes.

## 3. Results

### 3.1. General Characteristics of the Study Subjects

**Figure 1:**
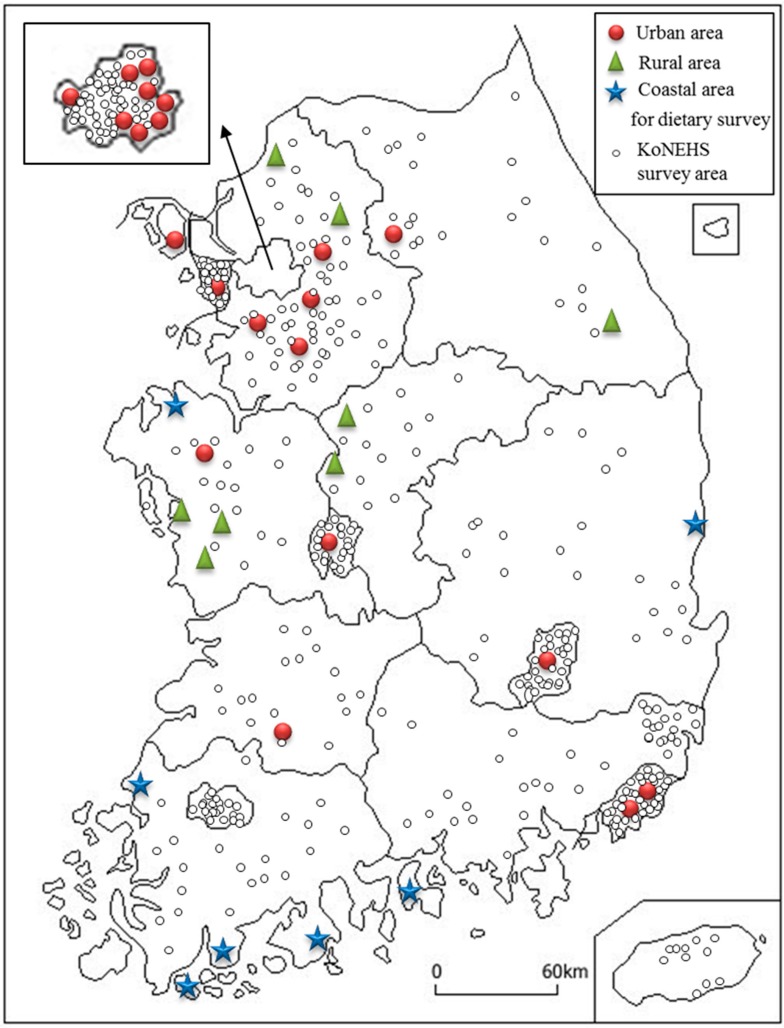
The geographic location of the pilot study area. Red circles, green triangles, and blue stars represent urban, rural, and coastal areas, respectively. Black open circles represent the total Korean National Environmental Health Survey (KoNEHS) target areas.

The dietary survey was conducted in 36 locations: 21 urban, 8 rural, and 7 coastal areas (Figure 1). These areas were selected from 400 KoNEHS target areas to be representative of the regional and seasonal distribution. There were no significant differences in the demographic characteristics between the selected subsample areas for the dietary survey and the total KoNEHS target areas.

The general characteristics of the study subjects are presented in Table 1. Males comprised about 44% of the total sample. There was a statistically significant different distribution of male and female subjects in smoking and alcohol drinking status (*p* < 0.001), while no differences were observed between genders for residential area, age group, and obesity status.

### 3.2. Daily Total and Methylmercury Intakes

The daily total mercury intakes of the subjects were estimated from the three-day food records, and the daily methylmercury intakes from fish and shellfish were calculated with a conversion shown in Equation (2). Table 2 shows the average daily intake of total mercury and methylmercury and the percentage of the PTDI by sex, age, residential area, obesity, smoking status, and alcohol drinking status. The subjects living in coastal areas had a higher intake of total mercury and methylmercury than those living in urban or rural areas (*p* < 0.001). The proportion of subjects whose inorganic mercury intake exceeded the PTDI was 0.18% (*n* = 1), whereas the proportion of subjects whose methylmercury intake from fish and shellfish exceeded the PTDI was about 3% (*n* = 16) of the total study subjects. The intakes of mercury and methylmercury were below the PTDI in most of the Korean population. The proportion of subjects whose intake of methylmercury exceeded the PDTI was higher in coastal areas (6.31%) than in urban (1.26%) or rural (4.00%) areas (*p* = 0.012). There were no significant differences according to obesity status and alcohol drinking status.

### 3.3. The Blood Mercury Concentration

Table 3 shows the blood mercury concentration and the proportion of subjects whose blood mercury level exceeded the HBM I value. The geometric means and 95% CIs of blood mercury in males (*n* = 241), females (*n* = 312), and the total subjects (*n* = 553) were 3.92 (3.64–4.23), 2.61 (2.46–2.77), and 3.12 (2.96–3.28) μg/L, respectively. The geometric mean of the blood mercury concentration of subjects living in coastal areas (4.36 μg/L) was significantly higher than those in urban (2.98 μg/L) or rural (2.59 μg/L) areas (*p* < 0.001). There was a significant difference in the blood mercury concentration by smoking and alcohol drinking status (*p* < 0.001).

The proportion of the study population with a high blood mercury level was significantly higher in coastal areas (34.55%) than in urban (17.72%) or rural (11.20%) areas (*p* < 0.001). Additionally, current alcohol drinkers were significantly more likely to have a high blood level of mercury than that of ex-drinkers or non-drinkers (*p* < 0.001).

### 3.4. The Association between the Dietary Mercury Intake and the Blood Mercury Level

The geometric mean of the blood mercury concentration increased significantly and linearly with the total mercury intake from all food items and the methylmercury intake from fish and shellfish (Table 4).

The odds ratios for the blood mercury levels above the HBM I value in the highest mercury and methyl mercury intake group were 3.27 (95% CI 1.79–5.95) and 3.20 (95% CI 1.77–5.79) times higher than that of the lowest intake group, respectively (Table 4).

### 3.5. Contribution of Different Food Groups to Mercury Intake

Table 5 shows the contribution of 18 food groups to the total mercury intake and the geometric mean of blood mercury concentration according to the amount of each food group consumed. The food group that contributed most to the mercury intake was fish accounting for 65.8% of mercury intake, followed by shellfish and other seafood, and grains, which contributed about 12% and 10% of mercury intake, respectively. The geometric mean of the blood mercury concentration increased significantly with increased consumption of fish, shellfish, and beverages and alcohol (Table 5). The odds ratios for the blood mercury levels above the HBM I value in the highest fish intake group, the highest shellfish and other seafood intake group and the highest beverages and alcohol intake group were 3.11 (95% CI 1.69–5.75), 3.28 (95% CI 1.77–6.05) and 2.95 (95% CI 1.57–5.56) times higher than that of the lowest intake group, respectively.

### 3.6. The Dose–Response Relationship between Seafood Intake and Blood Mercury Concentration

Table 6 shows the dose–response relationship between seafood intake, which was divided into fish and shellfish intake, and blood mercury concentration. The geometric mean of the blood mercury concentration increased significantly and linearly with both fish and shellfish intake per day. The odds ratio for blood mercury levels above the HBM I value of people who consumed ≥ 2 portions of fish per day were 2.07 (95% CI 1.15–3.72) times higher than people consuming < 1 portion of fish per day. This significant difference was not observed in analysis of shellfish and other seafood.

## 4. Discussion

In this study, dietary mercury intake of the Korean population was quantitatively estimated, and the association between dietary exposure and the blood mercury level was analyzed. The results show that the intakes of mercury and methylmercury were below the PTWI in most of the Korean population, and the blood mercury level in males and people living in coastal areas was relatively high; in addition, blood mercury concentration significantly and linearly increased with the mercury and methylmercury intake, especially from fish and shellfish. This study also found higher blood mercury levels among current drinkers versus ex- and non-drinkers.

The geometric mean of the blood mercury concentration in the study population was lower (3.12 μg/L) than those in other Korean studies (3.80 μg/L) [18], but greater than in the general population of European countries (0.58 μg/L) [26] or the United States (0.83 μg/L) [27].

In the general population, the total blood mercury concentration is related mostly to the dietary intake of organic forms of mercury, particularly methylmercury [28], which is known to be influenced by recent seafood consumption [29]. Thus, the high levels of blood mercury among Koreans may be related to their diet because of the high accessibility of the ocean. Moreover, our study showed fish and shellfish were the food groups that contributed most to dietary mercury intake, accounting for about 78% of the total mercury intake, and blood mercury level increased as the consumption of fish and shellfish increased.

However, there has been debate about the health effects of fish and shellfish with regard to the health benefits. Although fish and shellfish are the main sources of methylmercury intake, the beneficial health effects of these foods should also be considered so as not to focus exclusively on the harmful effects. Fish is regarded as a nutritious and healthy food because it is a source of high-quality proteins and omega-3 polyunsaturated fatty acid (PUFA), especially eicosapentaenoic acid (EPA) and docosahexaenoic acid (DHA). There is scientific evidence demonstrating that fish-derived PUFA has a positive association with fetal development, cardiovascular function, and prevention of Alzheimer’s disease [30]. These omega-3 fatty acids cannot be efficiently synthesized in our bodies; thus, it is necessary to obtain them in the diet through fish and fish-oil products [31]. Some nutrients found in fish, such as vitamin E and selenium, also have beneficial roles to offset the toxic effects of methylmercury [32]. Therefore, a cautious approach that takes into account both the toxic and beneficial effects of fish should be considered when determining how much fish to consume.

Importantly, other foods, including rice grown in contaminated fields, may contribute to mercury intake. Recent studies have reported that rice in some regions of China contains high levels of mercury due to contaminated soil and has become a significant source of mercury [33]. In our study, grains were ranked as the food group contributing the third highest amount of mercury, constituting 10% of the total mercury intake. Grains, especially rice, are the staple food of Korea. However, there was no significant association between grain consumption and blood mercury concentration. According to previous research, mercury concentration in rice is relatively low (0.0016 mg/kg in white rice, and 0.0025 mg/kg in brown rice) in Korea [34]; thus, rice consumption is not currently considered as a risk factor for mercury exposure.

Interestingly, alcohol consumption had a positive relationship with the blood mercury level in this study. Park and Lee also reported a strong positive association between alcohol consumption and blood mercury levels in a Korean adult population [19]. However, in another Korean cross-sectional study, blood mercury concentration was greater in abstainers (4.58 μg/L) than in drinkers (3.41 μg/L) [18]. A few studies have found that alcohol consumption may inhibit mercury absorption; however, only limited evidence is available [35]. Thus, further research into the association between alcohol consumption and mercury absorption is needed.

In our study, the proportion of subjects whose blood mercury level was above the HBM I value was about 20%, rising up to 35% in coastal areas. It is reasonable to assume that people living in coastal areas consume more fish and shellfish than people living in urban or rural areas. Because the HBM I value is defined as the concentration below which no adverse health effects are expected, people whose blood mercury level is above the value are not necessarily at risk. The number of people whose blood mercury level was above the HBM II (15 μg/L) value, which does indicate an increased risk of adverse health effects, was only 4 (0.7%).

In general, blood mercury concentration varies according to the demographic factor and health related behavior such as sex, residential area, and smoking status. To control these potential confounders, we adjusted for sex, age, smoking status, and drinking status in all analysis. However, there would be a possibility of interaction among independent variables, for example sex and smoking status, which can influence on the indicator of health such as blood mercury concentration [36]. In the result of additional analysis to test the interaction between sex and smoking status, there was no statistically significant interaction between sex and smoking status (*p* value for interaction = 0.794 in multiple regression model).

The strength of this study is its methodology, which enabled us to accurately estimate individual daily dietary intake. Previous studies that estimated dietary mercury exposure were limited in the accuracy of their estimations by using food frequency questionnaire methods, which could only measure the frequency of selected food items. However, in this study, we conducted a dietary survey using an open-ended dietary assessment method (three-day food record) and linked the food consumption dietary survey data to a mercury database to estimate the individual daily mercury intake. Once a database for a target hazardous material is constructed, any exposure through diet can be assessed quantitatively. To the best of our knowledge, this study is the first to examine the relationship between dietary mercury intake and the blood mercury levels in a large population in Korea. Furthermore, the accuracy of the final estimation was reasonable because we used a mercury database that covered most (about 94%) of the food groups consumed by the subjects of this study. It will be possible to estimate dietary mercury intake even more accurately by updating the database.

This study has some limitations. First, because of the cross-sectional design of the KoNEHS, we cannot confirm causation, but can make inferences about causal associations based on existing biological rationale. The biological half-life of methylmercury varied from 35 to 189 days with an average of 72 days [37]; thus, theoretically, present mercury concentration in blood reflected the diet several months ago. It would be a limitation of this study because dietary intake was assessed one week before the blood sample collection. However, it is known that recent seafood consumption would immediately raise the blood mercury levels [38]. In addition, three-day food record method is known to be the most accurate dietary assessment method for estimating usual diet of respondents. Therefore, time lag between dietary assessment and the blood collection of this study would be compensated. Furthermore, there was a question asking whether the diet as recorded was similar to usual diet of respondent. We found that most of the subjects of this study answered that there was no difference between recorded contents and their usual diet except 21 (3.8%). Therefore, it can be concluded that dietary mercury intake leads to an increase in the blood mercury level. Second, because the mercury database used to estimate dietary mercury intake was constructed based on the mean mercury content in food, the estimates produced in this study refer to the average content of food. In nature, the mercury content in food items varies extensively according to the area of cultivation, type of fish, and production. Therefore, the value estimated by linking with the database may not cover the full range of exposures (from minimum to maximum). If we update the mercury database by adding the minimum and maximum mercury contents of each food item, it will be possible to estimate the range of exposures according to the degree of mercury contamination in each food item. Third, mercury intake in this study would be underestimated if the subject did not eat some food containing high mercury during three days, but does eat these foods rarely or irregularly. Finally, the subjects in our study were adults (over 19 years of age); thus, a potentially vulnerable group (under 19 years of age) was not included. Children are highly vulnerable to toxic chemicals because of their lifecycle characteristics, and therefore, studies on the dietary mercury exposure of children are needed.

## 5. Conclusions

The results of this study provide compelling evidence that the blood mercury level has a strong positive association with dietary mercury intake, with fish and shellfish contributing more to mercury exposure than any other food group. We also found that the daily dietary exposure to mercury and the blood mercury level is considered safe in most of the population; however, people living in coastal areas have a relatively high intake of mercury and, consequently, a high blood mercury level. Because our study provides a framework to assess the dietary exposure to mercury, we expect further application of this study to the risk assessment of other hazardous materials for which dietary exposure is important.

## Figures and Tables

**Table 1:** General characteristics of the study population by classification variable.

Variables	Total		Male		Female		*p* Value ^1^
	*n*	%	*n*	%	*n*	%	
**Age (years)**							
20–29	37	6.69	16	6.64	21	6.73	NS
30–39	87	15.73	41	17.01	46	14.74	
40–49	115	20.80	41	17.01	74	23.72	
50–59	117	21.16	58	24.07	59	18.91	
60–69	122	22.06	52	21.58	70	22.44	
≥70	75	13.56	33	13.69	42	13.46	
**Residence ^2^**							
Urban area	317	57.32	135	56.02	182	58.33	NS
Rural area	125	22.60	57	23.65	68	21.79	
Coastal area	111	20.07	49	20.33	62	19.87	
**Obesity ^3^**							
Underweight	10	1.81	3	1.24	7	2.24	NS
Normal	328	59.31	135	56.02	193	61.86	
Obese	215	38.88	103	42.74	112	35.90	
**Smoking status ^4^**							
Current smoker	106	19.17	92	38.17	14	4.49	<0.001
Ex-smoker	89	16.09	85	35.27	4	1.28	
Non-smoker	358	64.74	64	26.56	294	94.23	
**Alcohol drinking status ^5^**							
Current drinker	325	58.77	181	75.10	144	46.15	<0.001
Ex-drinker	31	5.61	25	10.37	6	1.92	
Non-drinker	197	35.62	35	14.52	162	51.92	
**Total**	553		241	43.58	312	56.42	

NS: Non-Significant; ^1^ *p* values were calculated by chi-square test; ^2^ “Urban area” meant an administrative district such as “Dong”, “Rural area” meant an administrative district such as “Eup” or “Myeon”, and “Coastal area” meant an administrative district adjacent to the coast; ^3^ “Underweight” meant Body mass index (BMI) ≤ 18.5 kg/m^2^, “Normal” meant 18.5 kg/m^2^ < BMI ≤ 25 kg/m^2^, and “Obese” meant BMI ≥ 25 kg/m^2^; ^4^ “Current” meant have smoked any cigarette over his/her lifetime and still smokes, “Ex-” meant have smoked any cigarette over his or her lifetime and does not smoke at present; ^5^ “Current” meant had more than a drink in his/her lifetime and still drinks alcohol, “Ex-” meant had more than a drink in his/her lifetime and does not drink alcohol at present.

**Table 2:** Average daily intake of total mercury, methylmercury and percent PTDI.

		Total Mercury Intake (μg/day)	% PTDI for Inorganic Mercury ^1^	Methylmercury Intake from Fish and Shellfish (μg/day) ^2^	% PTDI for Methylmercury ^3^	% PTDI ≥ 100% for Inorganic Mercury			% PTDI ≥ 100% for Methylmercury		
	*n*	Mean ± SD	Mean ± SD	Mean ± SD	Mean ± SD	*n*	%	*p* Value ^4^	*n*	%	*p* Value ^4^
**Sex**											
Male	241	4.74 ± 6.83 ^a^	12.03 ± 17.44 ^a^	3.72 ± 6.60 ^a^	23.67 ± 41.99 ^a^	1	0.41	NS	10	4.15	NS
Female	312	3.07 ± 3.43 ^b^	9.16 ± 9.67 ^b^	2.19 ± 3.29 ^b^	16.27 ± 23.11 ^b^	0	0.00		6	1.92	
**Age (years)**											
20–29	37	2.33 ± 2.08 ^a^	6.74 ± 7.18 ^a^	1.62 ± 2.02 ^a^	12.04 ± 17.14 ^a^	0	0.00	NS	0	0.00	NS
30–39	87	3.26 ± 2.69 ^a^	8.51 ± 6.92 ^a^	2.35 ± 2.51 ^a^	15.19 ± 16.23 ^a^	0	0.00		0	0.00	
40–49	115	3.70 ± 4.45 ^a,b^	10.09 ± 12.30 ^a,b^	2.75 ± 4.29 ^a,b^	18.74 ± 29.56 ^a,b^	0	0.00		5	4.35	
50–59	117	4.13 ± 4.12 ^a,b^	11.31 ± 11.34 ^a,b^	3.12 ± 3.95 ^a,b^	21.33 ± 27.19 ^a,b^	0	0.00		4	3.42	
60–69	122	3.59 ± 4.68 ^a,b^	10.22 ± 12.73 ^a,b^	2.63 ± 4.50 ^a,b^	18.68 ± 30.57 ^a,b^	0	0.00		3	2.46	
≥70	75	5.11 ± 9.97 ^b^	13.86 ± 24.28 ^b^	4.16 ± 9.63 ^b^	27.80 ± 58.78 ^b^	1	1.33		4	5.33	
**Residence ^5^**											
Urban area	317	3.12 ± 3.20 ^a^	8.57 ± 8.74 ^a^	2.20 ± 3.05 ^a^	15.08 ± 20.81 ^a^	0	0.00	NS	4	1.26	0.012
Rural area	125	3.53 ± 4.09 ^a^	9.75 ± 11.37 ^a^	2.55 ± 3.93 ^a^	17.49 ± 27.32 ^a^	0	0.00		5	4.00	
Coastal area	111	6.04 ± 9.17 ^b^	16.43 ± 22.94 ^b^	5.08 ± 8.83 ^b^	34.37 ± 55.28 ^b^	1	0.90		7	6.31	
**Obesity ^6^**											
Underweight	10	2.67 ± 2.17	10.49 ± 8.47	2.02 ± 2.12	19.79 ± 20.72	0	0.00	NS	0	0.00	NS
Normal	328	3.64 ± 4.06	10.79 ± 12.03	2.71 ± 3.89	20.03 ± 28.84	0	0.00		12	3.66	
Obese	215	4.09 ± 6.77	9.84 ± 16.05	3.12 ± 6.53	18.66 ± 38.70	1	0.47		4	1.86	
**Smoking status ^7^**											
Current smoker	106	4.50 ± 4.73 ^a^	11.84 ± 13.80 ^a,b^	3.55 ± 4.56 ^a^	23.49 ± 33.11 ^a,b^	0	0.00	NS	6	5.66	0.002
Ex-smoker	89	5.82 ± 9.94 ^b^	14.78 ± 24.68 ^a^	4.76 ± 9.61 ^a^	30.21 ± 59.59 ^a^	1	1.12		6	6.74	
Non-smoker	358	3.09 ± 3.19 ^c^	8.91 ± 8.67 ^b^	2.18 ± 3.03 ^b^	15.65 ± 20.63 ^b^	0	0.00		4	1.12	
**Alcohol drinking status ^8^**											
Current drinker	325	4.18 ± 6.19	10.97 ± 15.50	3.23 ± 5.97	21.07 ± 37.36	0	0.00	NS	10	3.08	NS
Ex-drinker	31	3.95 ± 3.85	9.92 ± 9.59	2.95 ± 3.63	18.55 ± 22.52	0	0.00		1	3.23	
Non-drinker	197	3.15 ± 3.40	9.57 ± 10.68	2.23 ± 3.26	17.05 ± 25.49	1	0.31		5	2.54	
**Total**	553	3.80 ± 5.26	10.41 ± 13.67	2.86 ± 5.06	19.50 ± 32.88	1	0.18		16	2.89	

PTDI: Provisional Tolerable Daily Intake; SD: Standard Distribution; ^1^ The PTDI for inorganic mercury is 0.5714 μg/kg bw/day; ^2^ Methylmercury intake was calculated with the following equation: $\left[\mathrm{Methylmercury}\;\left(\mu g/g\right)\;in\;fish\right]=0.9\;\times \;\left[Total\;mercury\;\left(\mu g/g\right)\;in\;fish\right]\;\times \;\frac{M_{MeHg}\;\left(215.62\;g/mol\right)}{M_{THg}\;\left(200.59\;g/mol\right)}$; ^3^ The PTDI for methylmercury is 0.2286 µg/kg bw/day; ^4^ The *p* value was calculated by fisher’s exact test, NS: non-significant; ^5^ “Urban area” meant an administrative district such as “Dong”, “Rural area” meant an administrative district such as “Eup” or “Myeon”, and “Coastal area” meant an administrative district adjacent to the coast; ^6^ “Underweight” meant BMI ≤ 18.5 kg/m^2^, “Normal” meant 18.5 kg/m^2^ < BMI ≤ 25 kg/m^2^, and “Obese” meant BMI ≥ 25 kg/m^2^; ^7^ “Current” meant have smoked any cigarette over his/her lifetime and still smokes, “Ex-” meant have smoked any cigarette over his or her lifetime and does not smoke at present; ^8^ ”Current” meant had more than a drink in his/her lifetime and still drinks alcohol, “Ex-” meant had more than a drink in his/her lifetime and does not drink alcohol at present; ^a–c^ Duncan’s multiple range test was carried out for a post-hoc test within the column.

**Table 3:** The geometric mean of the blood mercury concentration and the percentage of people with blood mercury concentration ≥ 5 µg/L (HBM I value).

	Blood Mercury (μg/L) ^1^		Blood Mercury ≥ 5 μg/L ^2^		
	GM (95% CI)	*p* Value ^3^	*n*	%	*p* Value ^4^
**Sex**					
Male	3.92 (3.64–4.23)	<0.001	79	32.92	<0.001
Female	2.61 (2.46–2.77)		29	9.32	
**Age (years)**					
20–29	2.56 (2.18–3.00)	NS	4	10.81	NS
30–39	2.87 (2.54–3.25)		15	17.24	
40–49	3.14 (2.86–3.44)		20	17.39	
50–59	3.32 (2.99–3.68)		28	24.14	
60–69	3.05 (2.73–3.41)		21	17.36	
≥70	3.51 (2.94–4.20)		20	26.67	
**Residence ^5^**					
Urban area	2.98 (2.80–3.18)	<0.001	56	17.72	<0.001
Rural area	2.59 (2.34–2.87)		14	11.20	
Coastal area	4.36 (3.91–4.87)		38	34.55	
**Obesity ^6^**					
Underweight	2.35 (1.56–3.53)	NS	1	10.00	NS
Normal	3.02 (2.83–3.22)		62	18.96	
Obese	3.31 (3.05–3.59)		45	21.03	
**Smoking status ^7^**					
Current smoker	3.54 (3.17–3.96)	<0.001	30	28.30	<0.001
Ex-smoker	4.29 (3.80–4.85)		32	36.36	
Non-smoker	2.77 (2.61–2.94)		46	12.89	
**Alcohol drinking status ^8^**					
Current drinker	3.52 (3.30–3.76)	<0.001	88	27.08	<0.001
Ex-drinker	3.19 (2.54–4.00)		4	13.33	
Non-drinker	2.54 (2.35–2.74)		16	8.16	
**Total**	3.12 (2.96–3.28)		108	19.60	

GM: Geometric mean; CI: Confidence Interval; NS: Non-Significant; ^1^ Blood mercury concentration data were missing for two subjects; ^2^ The Human BioMonitoring (HBM) I value for blood mercury is 5 μg/L; ^3^ The *p* value was calculated by generalized linear model analysis; ^4^ The *p* value was calculated by chi-square test; ^5^ “Urban area” meant an administrative district such as “Dong”, “Rural area” meant an administrative district such as “Eup” or “Myeon”, and “Coastal area” meant an administrative district adjacent to the coast; ^6^ “Underweight” meant Body mass index (BMI) ≤ 18.5 kg/m^2^, “Normal” meant 18.5 kg/m^2^ < BMI ≤ 25 kg/m^2^, and “Obese” meant BMI ≥ 25 kg/m^2^; ^7^ “Current” meant have smoked any cigarette over his/her lifetime and still smokes, “Ex-” meant have smoked any cigarette over his or her lifetime and does not smoke at present; ^8^ “Current” meant had more than a drink in his/her lifetime and still drinks alcohol, “Ex-” meant had more than a drink in his/her lifetime and does not drink alcohol at present.

**Table 4:** The geometric means of the blood mercury concentration and the odds ratios for blood mercury ≥ the HBM I value according to the total mercury and methylmercury intakes.

		Dietary Intake			Blood Mercury (μg/L) ^1^		*p* Value ^2^	OR for Blood Mercury ≥ HBM I ^3^	
		*n*	Mean	Range	GM	95% CI		OR	95% CI
Total mercury intake (μg/day)	T1	184	0.99	(0.32–1.48)	2.56	(2.35–2.78)	<0.001	1.00	-
	T2	185	2.42	(1.49–3.56)	2.84	(2.62–3.08)		1.29	(0.67–2.50)
	T3	184	7.99	(3.57–81.72)	4.16	(3.84–4.51)		3.27	(1.79–5.95)
% PTDI for inorganic mercury ^4^	T1	184	2.73	(0.79–4.18)	2.60	(2.40–2.83)	<0.001	1.00	-
	T2	185	6.77	(4.19–9.99)	3.02	(2.77–3.30)		1.53	(0.81–2.88)
	T3	184	21.77	(10.00–195.91)	3.85	(3.55–4.16)		3.29	(1.81–6.01)
Methylmercury intake from fish and shellfish (μg/day) ^5^	T1	184	0.20	(0–0.63)	2.56	(2.35–2.79)	<0.001	1.00	-
	T2	185	1.52	(0.64–2.57)	2.88	(2.65–3.14)		1.30	(0.67–2.51)
	T3	184	6.86	(2.58–78.27)	4.09	(3.79–4.42)		3.20	(1.77–5.79)
% PTDI for methylmercury ^6^	T1	184	1.41	(0–4.71)	2.56	(2.35–2.78)	<0.001	1.00	-
	T2	185	10.62	(4.71–17.99)	3.01	(2.76–3.28)		1.54	(0.81–2.94)
	T3	184	46.51	(18.00–469.06)	3.94	(3.64–4.26)		3.13	(1.72–5.67)

GM: Geometric Mean; CI: Confidence Interval; OR: Odds Ratio; PTDI: Provisional Tolerable Daily Intake; T: Tertile; ^1^ Blood mercury concentration data were missing for two subjects; ^2^ The *p* values were calculated by generalized linear model analysis and adjusted for sex and age; ^3^ The HMB I value for blood mercury is 5 μg/L, and the odds ratio was adjusted for sex, age, smoking status, and drinking status; ^4^ The PTDI for inorganic mercury is 0.5714 μg/kg bw/day; ^5^ The methylmercury intake was calculated with the following equation: $\left[\mathrm{Methylmercury}\;\left(\mu g/g\right)\;in\;fish\right]=0.9\;\times \;\left[Total\;mercury\;\left(\mu g/g\right)\;in\;fish\right]\;\times \;\frac{M_{MeHg\;}\left(215.62\;g/mol\right)}{M_{THg}\;\left(200.59\;g/mol\right)}$; ^6^ The PTDI for methylmercury is 0.2286 μg/kg bw/day.

**Table 5:** Contribution rate to mercury intake, the blood mercury concentration and Odds Ratios for blood mercury ≥ the HBM I value according to the food group intake.

Rank	Food Group	Average Food Group Intake (g/day)	Average Mercury Intake (μg/day)	Contribution Rate (%)	Blood Mercury (μg/L) GM (95% CI) ^1^				OR for Blood Mercury ≥ HBM I ^3^			
					T1	T2	T3	*p* Value ^2^	T1	T2	T3	*p* Value
1	Fish	47.3	2.50	65.8	2.55 (2.35–2.77)	2.99 (2.75–3.24)	3.97 (3.64–4.32)	<0.001	1.00	1.73 (0.90–3.32)	3.11 (1.69–5.75)	<0.001
2	Shellfish and other Seafood	65.3	0.45	12.0	2.53 (2.32–2.76)	3.00 (2.78–3.25)	3.98 (3.65–4.33)	<0.001	1.00	1.87 (0.97–3.58)	3.28 (1.77–6.05)	<0.001
3	Grains	263.1	0.38	10.0	3.06 (2.81–3.34)	3.28 (3.00–3.59)	3.01 (2.76–3.28)	NS	1.00	0.50 (2.80–0.91)	0.62 (0.35–1.11)	NS
4	Vegetables	281.5	0.16	4.3	2.81 (2.59–3.06)	3.10 (2.83–3.39)	3.47 (3.19–3.78)	NS	1.00	0.77 (0.43–1.40)	1.07 (0.59–1.92)	NS
5	Fruits	166.9	0.07	1.8	3.26 (2.97–3.58)	3.10 (2.86–3.37)	2.66 (2.75–3.26)	NS	1.00	0.84 (0.48–1.47)	0.86 (0.48–1.53)	NS
6	Meats	74.0	0.05	1.2	3.01 (2.75–3.29)	3.17 (2.89–3.47)	3.18 (2.94–3.44)	NS	1.00	1.49 (0.81–2.74)	1.60 (0.85–2.99)	NS
7	Mushrooms	4.0	0.04	1.1	3.22 (3.02–3.43)	2.59 (2.12–3.18)	3.02 (2.76–3.29)	NS	1.00	0.70 (0.19–2.56)	1.11 (0.68–1.81)	NS
8	Beverages and Alcohols	179.5	0.03	0.8	2.85 (2.62–3.10)	2.99 (2.77–3.23)	3.56 (3.23–3.91)	0.002	1.00	1.27 (0.66–2.44)	2.95 (1.57–5.56)	<0.001
9	Legumes	41.5	0.03	0.8	3.05 (2.81–3.32)	3.19 (2.91–3.50)	3.11 (2.85–3.38)	NS	1.00	1.30 (0.74–2.28)	0.84 (0.47–1.51)	NS
10	Seaweeds	3.7	0.02	0.6	3.07 (2.80–3.37)	2.87 (2.66–3.11)	3.43 (3.14–3.74)	0.029	1.00	0.69 (0.38–1.24(	1.11 (0.64–1.92)	NS
11	Potatoes and Starch	32.6	0.02	0.5	3.26 (2.97–3.59)	3.03 (2.79–3.30)	3.06 (2.82–3.32)	NS	1.00	0.88 (0.50–1.54)	1.10 (0.62–1.94)	NS
12	Eggs	20.5	0.01	0.4	3.25 (2.96–3.56)	3.08 (2.82–3.37)	3.03 (2.79–3.28)	NS	1.00	1.07 (0.61–1.89)	0.92 (0.51–1.67)	NS
13	Seasonings	35.9	0.01	0.3	2.72 (2.49–2.98)	3.12 (2.86–3.40)	3.56 (3.28–3.87)	NS	1.00	1.24 (0.68–2.26)	1.64 (0.91–2.93)	NS
14	Milk and Dairy Products	58.4	0.01	0.3	3.29 (3.06–3.53)	2.89 (2.58–3.22)	2.97 (2.72–3.25)	NS	1.00	0.44 (0.20–0.98)	0.92 (0.54–1.55)	NS
15	Others	2.3	0.00	0.1	3.09 (2.93–3.26)	3.31 (2.90–3.79)	-	NS	-	1.00	0.90 (0.44–1.86)	NS
16	Fat and Oils	7.3	0.00	0.0	3.08 (2.81–3.37)	3.21 (2.94–3.52)	3.06 (2.83–3.32)	NS	1.00	1.65 (0.94–2.90)	1.23 (0.67–2.24)	NS
17	Sugars	6.5	0.00	0.0	3.14 (2.86–3.43)	3.24 (2.96–3.54)	2.98 (2.75–3.23)	NS	1.00	0.91 (0.52–1.61)	1.21 (0.68–2.13)	NS
18	Seeds and Nuts	4.4	0.00	0.0	3.23 (2.95–3.54)	3.07 (2.82–3.35)	3.05 (2.80–3.31)	NS	1.00	1.04 (0.59–1.83)	0.98 (0.56–1.72)	NS

GM: Geometric Mean; CI: Confidence Interval; OR: Odds Ratio; NS: Non-Significant; T: Tertile; ^1^ Blood mercury concentration data were missing for two subjects; ^2^ The *p* values were calculated by generalized linear model analysis and adjusted for sex and age; ^3^ The HBM I value for blood mercury is 5 µg/L and the odds ratio was adjusted for sex, age, smoking status, and drinking status.

**Table 6:** The dose-response relationship between seafood intake and blood mercury concentration.

	Fish (Portion Size/Day) ^1^				Shellfish and Other Seafood (Portion Size/Day) ^1^			
	<1	1–2	≥2	*p* Value ^3^	<1	1–2	≥2	*p* Value ^4^
*n* (%)	317 (57.3)	140 (25.3)	96 (17.4)	<0.001	514 (93.0)	29 (5.2)	10 (1.8)	<0.001
Food group intake (g/day) [Mean ± SD]	14.3 ± 13.0	53.4 ± 21.0	147.4 ± 125.3	<0.001	56.2 ± 74.8	167.4 ± 88.7	237.3 ± 46.6	<0.001
Methylmercury intake (μg/day) [Mean ± SD]	0.6 ± 0.9	2.5 ± 1.7	8.1 ± 9.5	<0.001	0.3 ± 0.5	2.3 ± 1.0	3.9 ± 1.4	<0.001
Blood mercury (μg/L) ^2^ [GM (95% CI)]	2.77 (2.60–2.96)	3.44 (3.11–3.80)	3.96 (3.51–4.46)	0.001	3.07 (2.91–3.24)	3.66 (3.02–4.42)	4.19 (3.49–5.04)	0.030
OR for Blood mercury ≥ HBM I ^4^ [OR (95% CI)]	1.00	1.24 (0.72–2.15)	2.07 (1.15–3.72)	0.050	1.00	2.30 (0.95–5.58)	0.65 (0.08–5.56)	NS

GM: Geometric Mean; CI: Confidence Interval; OR: Odds Ratio; NS: Non-Significant; ^1^ The portion sizes of fish and shellfish was calculated based on the Dietary Reference Intakes for Koreans 2015 [25]; ^2^ Blood mercury concentration data were missing for two subjects; ^3^ The *p* values were calculated by generalized linear model analysis and adjusted for sex and age, smoking status, and drinking status; ^4^ The HBM I value for blood mercury is 5 μg/L and the odds ratio was adjusted for sex, age, smoking status, and drinking status.
